# Atypical presentation of adenosine deaminase 2 deficiency with bi‐allelic ADA2 mutation

**DOI:** 10.1002/ccr3.5408

**Published:** 2022-03-01

**Authors:** Reem Al‐shaikh, Dimah Alnowaiser, Abdul Ali Peer‐Zada, Awatif Almutairi, Hamza Alghamdi

**Affiliations:** ^1^ General Pediatrics Department, Allergy and Immunology Section Children's Specialized Hospital King Fahad Medical City Riyadh Saudi Arabia; ^2^ Molecular Pathology (Genetics) Section, Pathology and Clinical Laboratory Medicine Administration King Fahad Medical City Riyadh Saudi Arabia

**Keywords:** ADA2, autoinflammation, autosomal recessive vasculitis, hematologic defects syndrome, immunodeficiency

## Abstract

Herein, we report a case of VAIHS with atypical clinical presentation of perianal abscess, fistula fever, and bi‐cytopenia including pathogenic ADA2 mutation suggesting that ADA2 deficiency be considered as a differential diagnosis of enlarging cutaneous abscess with no evidence of wound healing in the setting of leukopenia and neutropenia.

## Graphical abstract

A challenging case of VAIHS with atypical presentation and ADA2 mutation highlights the consideration of ADA2 deficiency as a differential diagnosis of enlarging cutaneous abscess with no wound healing in the setting of leukopenia and neutropenia.

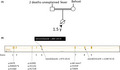

## 1 INTRODUCTION

Vasculitis, autoinflammation, immunodeficiency, and hematologic defects syndrome (VAIHS) or adenosine deaminase 2 deficiency (DADA2) is a rare autosomal recessive disease characterized by a highly variable clinical phenotype that is caused by bi‐allelic mutations in ADA2 (formerly known as Cat Eye Syndrome candidate region 1 or CECR1).^1^, ^2^ The manifestation of VAIHS varies greatly among children and adults, including those homozygous for the same founder mutation, in the form of varying ages of presentation and variability of symptoms and severity.^3^, ^4^, ^5^ Vasculitis, usually manifested before ten years of age, appears as ischemic/hemorrhagic strokes or cutaneous polyarteritis nodosa (PAN), which remains a predominant feature in most cases described to date.^6^ Autoinflammatory characteristics constitute periodic fevers, rashes (livedo racemose/ reticularis), and musculoskeletal involvement in the form of myalgia/arthralgia, arthritis, and myositis.^7^ Dysregulation of immune response may manifest as autoimmunity, bone marrow deficiencies, and lymphadenopathy with some patients presenting lymphoproliferative disease. Hematologic defects can include red cell aplasia, thrombocytopenia, neutropenia, or pancytopenia.^8^, ^9^ Gastrointestinal manifestations in the form of hepatosplenomegaly, abdominal pain, and inflammatory bowel disease can occur in up to 10% of patients. More severe manifestations in the form of neurological defects such as dysarthria, ataxia, cranial nerve palsies, cognitive impairment or kidney, and intestine injury may also occur.^10^, ^11^

**FIGURE 1:**
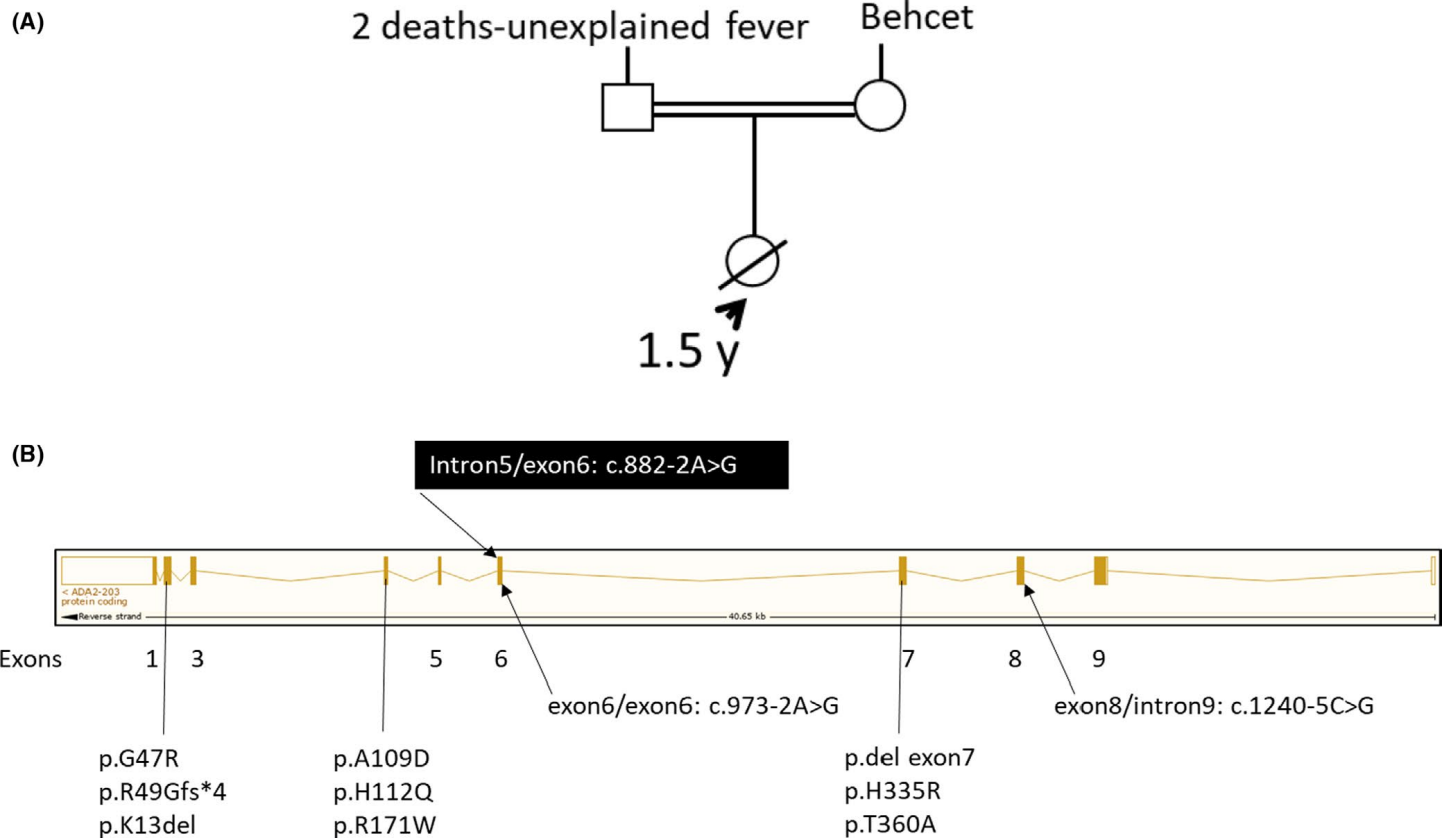
Family pedigree showing consanguinity and exon map of ADA2 gene showing various known variants including c.882‐2A>G variant identified in the current case (A) Family pedigree of the only child (affected) from consanguineous parents with a strong paternal and maternal family history. (B) ADA2 gene exons (solid bars, numbered 1–9) showing various known ADA2 variants in the designated exons or intron (lines and arrows) and c.882‐2A>G splicing variant (solid black box) identified in the current case within intron5/exon6 region

The diagnosis of VAIHS is established in individuals with clinical and laboratory features suggestive of DADA2, and the diagnosis is confirmed by the presence of bi‐allelic loss‐of‐function ADA2 variants and/or low (<5% of normal) or undetectable levels of ADA2 activity in plasma or serum. More than 60 disease‐causing ADA2 variants in VAIHS, mostly missense single nucleotide such as c.139G>A; p. Gly47Arg (Figure 1), and some splicing variants have been described across the entire coding region of ADA2.^3^, ^4^ The ADA2 locus contains variability among humans, resulting in polymorphic variations associated with DADA2.^3^ Depending upon the phenotypic variability in VAIHS, laboratory findings may include elevated C‐reactive protein (CRP), erythrocyte sedimentation rate (ESR), transaminases, absence of antineutrophilic cytoplasmic antibodies (ANCA), hypogammaglobulinemia with low levels of IgM, IgG, and/or IgA, positive lupus anticoagulant, pancytopenia and bone marrow hypo/hypercellularity, grade I myelofibrosis, lymphocyte infiltrate (predominantly CD8+), and mild reticulin fibrosis.^6^, ^10^, ^11^

The treatment of VAIHS in patients with bi‐allelic ADA2 pathogenic variants to relieve autoinflammatory/vasculitis manifestations reduces the risk of stroke and relieves immunodeficiency involves anti‐tumor‐necrosis factor (TNF) agents such as etanercept, adalimumab, golimumab, infliximab, and certolizumab, although anti‐TNF agents may not have any impact on severe bone marrow abnormalities.^8^, ^9^

The current case presented with atypical phenotypic features, and therefore, DADA2 was not considered. Whole‐exome sequencing was, however, requested to identify any likely genetic cause that revealed pathogenic ADA2 variant suggesting DADA2. The aim of this report is to highlight the consideration of ADA2 deficiency as a differential diagnosis in patients manifesting enlarging cutaneous abscess with no evidence of wound healing in the setting of leukopenia and neutropenia. This case of VAIHS patient presented at a very early age with a perianal abscess, fistula formation, fever, aphthous ulcers, bi‐cytopenia, and hematochezia.

## 2 CASE PRESENTATION

Patient X is the index case, previously healthy 18 months old woman, the first child of healthy Saudi consanguineous parents (Figure 1), presented to our Emergency Department with a five‐day history of high‐grade fever, associated with diarrhea development lethargy, and anal lesion. Fever reached a T‐max of 39°C and was partially responsive to antipyretics. Parents reported a strong family history from both paternal and maternal side. There were two childhood deaths at two years of age from fever of unknown origin (paternal side) with one family member requiring intravenous immunoglobulin and one family member with Bechet's disease (maternal side). Physical examination revealed a normal child with no findings apart from the presence of oral aphthous ulcers along buccal mucosa and anal lesion, described to be 1cm x 1cm regular erythematous painless papule. The child underwent diagnostic workup and the initial investigations showed neutropenia and leukopenia, with white blood count, WBC 4.58 x 109/L (reference range 4.3–11.3 109/L); ANC 0.13 x 109/L; hemoglobin 10 g/dl (reference range 11–15 g/dl); and platelet 154 × 109/L (reference range 155–435 × 109/L). Inflammatory markers showed increased CRP (148 mg/L, reference range 1–3 mg/L) and ESR (120, reference range 0–20). Blood, urine, and cerebrospinal fluid cultures were negative.

Throughout her hospital stay, fever persisted with measurements at four hourly intervals reaching a maximum of 40°C over three months. The anal lesion enlarged and was complicated by perianal fistula formation at 2 o'clock and 9 o'clock position associated with exudative discharge showing growth of *P*. *aeruginosa* and *E*. *coli*. Multiple aphthous ulcers developed into oral mucosa; oral scrapings showed growth of *C*. *tropicalis*. Diarrhea progressed to include hematochezia and blood clot passage requiring multiple blood transfusions.

The patient continued to show persistent neutropenia (0–0.35 × 103/µl, reference range 1.35–7.5 × 103/µl), lymphopenia (0.22–4.92 × 103/µl, reference range 1.9–4.9 × 103/µl) with the development of thrombocytopenia. Inflammatory markers remained elevated: CRP (310 mg/L, reference range 1–3 mg/L), ESR (120, reference range 0–20), and serum ferritin (15141 ng/ml, reference range 10–204 ng/ml), and the coagulation profile remained normal. Antinuclear antibodies (ANA) and anti‐double‐stranded DNA (anti‐dsDNA) were negative. Immunoglobulin levels IgG (3.87 g/L, reference range 2.9–10.7 g/L), IgA (0.28 g/L, reference range 0.40–1.20 g/L), and IgM (0.2 g/L, reference range 0.3–1.5 g/L) remained unaffected along with post‐vaccination titer. Oxidative burst test, extensive workup for virology, fungemia, and mycobacterium, and C3/C4 complement factors were all normal.

The perianal abscess was complicated by growing size; and failure to forming granulation tissue. Initially, stool for occult blood resulted positive, with the development of microcytic hypochromic anemia. Abscess depth progressed to involve vascular beddings resulting in hematochezia and fresh blood passage. Hemoglobin reached to 4 mg/dl, requiring multiple blood transfusions and fresh frozen plasma (FFP). The source of bleeding was not identified. Mickell's scan, RBC technetium scan, ultrasonography for intussusception and organomegaly, endoscopy, and colonoscopy findings from the rectum to ascending colon were all normal.

Intervention for neutropenia included methylprednisolone 10 mg/kg and intravenous immunoglobulin therapy, with no response. A granulocyte colony‐stimulating factor (G‐CSF) infusion of 200 units for one month was initiated, with a lack of response. Furthermore, HLA (Human Leukocyte Antigen) typing was performed on both parents for any future bone marrow transplantation (BMT).

Bone marrow aspiration showed hypocellularity (50% cellularity), myeloid aplasia, and extensive histiocytic proliferation with significant hemophagocytosis. Flow cytometry analyses on bone marrow aspirate showed severe lymphocytopenia encompassing all lymphocyte subsets (B‐, T‐, NK cells) suggesting primary or secondary immunodeficiency due to bone marrow suppression.

Monitoring of the daily blood passage revealed about 50 ml of the presence of clots. With continuous fever and the development of tachycardia reaching a heart rate of 200 bpm, the patient was admitted under the care of the Pediatric Intensive Care Unit (PICU). Despite multiple trials and continuous infusion, platelets failed to reach the acceptable safe level. Interleukin‐1 receptor agonist anakinra (10 mg/kg) was initiated after a multi‐disciplinary decision involving General Pediatrics, Immunology, Hematology‐Oncology, Infectious diseases, Gastroenterology, Rheumatology, Pediatric Surgery, Pathology, and PICU was reached. Fever subsided for two days and resumed. A chimeric monoclonal antibody, infliximab was given with no satisfactory outcome. Persistent massive peri‐rectal bleeding and tachycardia continued for a duration of two months. Cardiac ejection fraction was reduced, patient started on inotropes and supportive management. The patient developed acute kidney injury, liver injury with abdominal distention. Respiratory status declined secondary to abdominal compartment syndrome, and she was placed on maximum respiratory support. Despite all interventions, the patient was arrested. The genetic testing using whole‐exome sequencing came positive and revealed a homozygous pathogenic variant in ADA2 gene suggesting VAIHS.

## 3 DISCUSSION

We report a case with atypical clinical presentation of perianal abscess in the setting of leukopenia and neutropenia at disease onset in which whole‐exome sequencing revealed a homozygous pathogenic variant c.882‐2A>G in ADA2 gene suggesting VAIHS. The clinical description of this case not only emphasizes on the highly variable phenotype associated with ADA2 deficiency due to CECR1 mutations but also to the diagnostic limitations it may entail. VAIHS patients with highly pleiotropic clinical features may manifest only some of them, thereby making the diagnosis difficult in the event of non‐availability of single‐gene or multigene panel testing. The chance of missed diagnosis may even be exacerbated when atypical clinical features are manifested at disease onset, like our case. ADA2 deficiency was first described in two major articles, one in patients with fever, visceral and cutaneous lesions compatible with polyarteritis nodosa, and the other in patients with peripheral and central nervous system involvement.^2^, ^3^ While the patient in this report eventually demonstrated a comparable spectrum of disease features, she initially presented with a perianal abscess and persistent fever. Another diagnostic limitation in VAIHS patients is the non‐availability of clinically validated ADA2 enzyme assays, which limits the confirmation of diagnosis only to the molecular genetic testing with an extended turn‐around‐time (TAT) and thus, delaying the diagnosis.

ADA2 deficiency should be considered as a differential diagnosis of enlarging cutaneous abscess with no evidence of wound healing in the setting of leukopenia and neutropenia. Our patient exhibited no hypogammaglobulinemia, no biological markers of autoimmunity (ANA/lupus anticoagulant), and no recurrent infections, even though there was B‐cell lymphopenia in accordance with the proposed hypothesis that ADA2 deficiency may lead to a defect in memory B cells.^8^, ^11^ Various clinical manifestations of ADA2 deficiency (VAIHS, OMIM#615688) include vasculopathy, skin manifestations, neuropathy, immunodeficiency, and hematology.

Our patient showed a splicing variant c.882‐2A>G in ADA2 gene. ADA2 (Adenosine Deaminase 2, OMIM#607575) gene encodes a member of a subfamily of the adenosine deaminase protein family that catalyzes the deamination of adenosine and 2‐prime‐deoxyadenosine to inosine and deoxy‐inosine, respectively, and regulates levels of adenosine signaling, cell proliferation, and differentiation. Diseases associated with ADA2 include Sneddon syndrome (SNDNS, OMIM#182410), vasculitis, autoinflammation, immunodeficiency and hematologic defects syndrome (VAIHS, OMIM#615688), Kaposi sarcoma susceptibility, and Diamond‐Blackfan anemia 1. The variant c.882‐2A>G (ADA2, NM_001282225.2) is reported in ClinVar and Varsome databases as pathogenic for polyarteritis nodosa or VAIHS and has been previously described to resemble ALPS (autoimmune lymphoproliferative syndrome) like phenotype by Alsutlan et al. 2018 (PMID 29271561).^13^ There is no precise genotype‐phenotype correlation in the reported ADA2 mutations, and further studies are needed to evaluate the effects of this mutation on RNA splicing, stability, and translation. Variability in disease severity has been reported even among patients with the same mutation, as previously reported in individuals with homozygous p. Arg169Gln mutation, indicating potential genetic, epigenetic, and environmental factors in determining the severity of the phenotype.^3^, ^4^, ^5^, ^6^

The use of steroids, anakinra, infliximab, and G‐CSF was not effective in this condition. Studies have exhibited benefit from the use of anti‐TNF agents such as etanercept, adalimumab, and thalidomide resulting in complete remission.^8^ In addition, the use of fresh frozen plasma (FFP), considered a crucial therapy in DADA2, was also not beneficial, possibly due to the short half‐life of ADA2.^9^ Hematopoietic stem cell transplantation (HSCT) with its potential advantages in the cure of the disease and avoidance of long‐term cost and toxicity of lifelong anti‐TNF therapy^8^, ^9^, ^10^, ^14^ was an option, and therefore, HLA typing was done on both parents. However, the current case did not receive HSCT due to complications. HSCT was reported to treat DADA2 effectively.^14^

The classic DADA2 phenotype includes vasculitis, stroke, livedo reticularis, hepatosplenomegaly, hypogammaglobulinemia, and cytopenia, and there is high phenotypic variability in DADA2.^7^, ^12^, ^13^ Our patient had cutaneous vasculitis and leukopenia, and neutropenia without evidence of lacunar strokes, organomegaly, and dysregulation of immune response rendering diagnosis more challenging. Thus, screening for DADA2 should be considered in the differential diagnosis of children presenting with persistent fever, cytopenia, and non‐healing cutaneous lesions. Marked phenotypic variability in DADA2 can occur, and screening in families should be initiated in those with evidence of ADA2 mutation. Efforts to collect new cases may allow us to close potential diagnostic gaps, straddling the borders of autoinflammation, and immunological deficiency.^15^ Importantly, although the pathological basis of this severe disease remains unclear, highly promising therapeutic strategies have already emerged. In conclusion, this case suggests that ADA2 deficiency should be considered as a differential diagnosis in patients having enlarged cutaneous abscess with no evidence of wound healing in the setting of leukopenia and neutropenia.

## CONFLICT OF INTEREST

The authors have no conflict of interest disclosure.

## AUTHOR CONTRIBUTIONS

RA and DA collected all the patient information including laboratory data and wrote the case description. AAP wrote the molecular genetics part and edited the manuscript. AA and HA edited the clinical part of the manuscript and were involved in the clinical management of the patients.

## ETHICAL APPROVAL

The case report consent was approved by KFMC Institutional Review Board in accordance with research ethics principles.

## CONSENT

Written informed consent was obtained from the legal guardian of the patient to publish this report in accordance with the journal's patient consent policy.

## Data Availability

None.
