#  Corrigendum

**DOI:** 10.1002/ccr3.6068

**Published:** 2022-07-14

In the article entitled “Atypical presentation of adenosine deaminase 2 deficiency with bi‐allelic ADA2 mutation” which was previously published in Volume 10 Issue 3 of Clinical Case Reports, the corresponding author's email id was published incorrectly. It has been updated as hamalghamdi@kfmc.med.sa, in this version.
